# Exploring the Limitations of the Shielding Function of Categorization Rules in Task-Switching

**DOI:** 10.3389/fpsyg.2019.01212

**Published:** 2019-05-28

**Authors:** Dong Guo, Bingxin Li, Yun Yu, Xuhong Liu, Xiangqian Li

**Affiliations:** ^1^School of Social Development and Public Policy, Fudan University, Shanghai, China; ^2^School of Psychology, University of Glasgow, Glasgow, United Kingdom; ^3^Institute of Psychology, Chinese Academy of Sciences, Beijing, China; ^4^Institute of International and Comparative Education, East China Normal University, Shanghai, China

**Keywords:** task-switching, bivalent features, shielding function of task set, task-switching cost, congruency effect

## Abstract

Applying categorization rules narrows attention toward the relevant features of a target and helps participants to ignore the irrelevant features of the target. This is called the shielding function of categorization rules. Here we explored the limitation of the shielding function in two task-switching experiments. In Experiment 1, we assigned each target a single digital numeral as an additional feature in addition to conventional bivalent features as in the previous task-switching experiments with bivalent tasks. In the first two stages of Experiment 1, half of the participants learned the numeral-response associations and the other half used an alternative numeral-categorization rule to infer the response. Without participants applying conventional task-switching rules, the switching costs disappeared. Moreover, when participants performed tasks by numeral-response associations the bivalent features interfered with response retrieval and caused response-congruency effects, whereas when participants applied the numeral-categorization rule, the bivalent features were shielded away and thereby the response-congruency effects disappeared. In the third stage, in which all participants applied task-switching rules by discriminating between bivalent features (i.e., filling and orientations), we found task-switching costs and response-congruency effects. In Experiment 2, new bivalent features produced stronger interference compared to Experiment 1. As a consequence, participants in both the association group and the numeral-categorization rule group showed significant response-congruency effects in the first two stages, where task-switching rules were not introduced. It follows that the shielding function of categorization rules has limits—strong interference from bivalent features can break down the shielding function. In addition, participants in the association group showed task-switching costs without being informed about the task-switching rules. We propose that strong proactive interference can produce task-switching costs even without the use of task-switching rules.

## Introduction

One of the major functions of human cognitive control is to guide our focus of attention to relevant information and, ideally, ignore any irrelevant information or interference. In a series of studies, Dreisbach and colleagues successfully demonstrated that applying categorization rules can modulate the information selection process and help people ignore the interference (Dreisbach and Haider, 2008, 2009; Dreisbach and Wenke, 2011; Dreisbach, 2012; Reisenauer and Dreisbach, 2013, 2014; Bogon et al., 2017).

Dreisbach and Haider (2008) for the first time demonstrated the shielding function of the categorization rules in a task-switching paradigm with univalent targets. In their experiment, eight targets were German words in green or red. When applying conventional task-switching rules, participants needed either to decide whether a German word started with a consonant or vowel (phoneme task), or decide whether the word was an animal or not (animal task). The color of the words functioned as task cues. Importantly, the arrangement of the targets allowed participants to use two other alternative strategies apart from applying the task-switching rules. Participants were instructed either to remember all eight target-response associations directly or to apply a simple categorization rule. The categorization rule instructed participants to press the left key if the target referred to something that can move (e.g., a bug) and to press the right button if the target referred to something that cannot move (e.g., Ulm). If participants applied one of the alternative strategies, the color of the words was completely irrelevant to the response. However, Dreisbach and Haider (2008) reported that participants who applied the target-response associations still processed the irrelevant color feature of the words, as was indicated by a significant interaction between Response repetition effect (response-repeat, response-switch) and Color switching effect (repeat, switch): When the response was repeated (*trial n–1* and *trial n* had the same response), color switching delayed the responses, but when the response was switched, color switching facilitated the responses. In contrast, for participants who applied the move/non-move categorization rule, this interaction was completely absent.

These findings demonstrated the shielding function of categorization rules—that is, when participants apply a categorization rule, the attention is narrowed toward the relevant feature of the target (i.e., mobility), and this helps participants to ignore the irrelevant information (in this instance, and the colors). In contrast, when participants apply the strategy of target-response associations, any target features might be taken as relevant in order to guide a response and thus they cannot ignore the interference from irrelevant distractions.

Although the shielding function of categorization rules is well established, a related question has not been well discussed in previous studies: the limitation of the shielding function. In a study by Dreisbach and Haider (2009), the categorization rules cannot shield participants against interferences that were semantically related to the categorization rules. Nevertheless, the results of their study seem to suggest that as long as the categorization rule was semantically unrelated to the interference, it can always successfully shield interference away, no matter how strong this interference is. We believe that a more practical model of the shielding function of categorization rules should put the limitation of such shielding function into consideration. In other words, if the irrelevant information or the interference were too strong, the shielding function might no longer keep them away from the focus of attention. We aimed to explore the limitation of the shielding function of the categorization rules in the present study. In Experiment 1, we replicated the shielding effect of categorization rules with a filling/orientation task-cueing paradigm. In Experiment 2, we demonstrated the limitation of the shielding function of the categorization rules.

## Experiment 1

In Experiment 1, we sought to replicate the shielding effect of categorization rules (Dreisbach and Haider, 2008, 2009; Dreisbach and Wenke, 2011; Dreisbach, 2012; Reisenauer and Dreisbach, 2013, 2014; Bogon et al., 2017), using a filling/orientation task-cueing paradigm adapted from Li et al. (2019a, Experiment 2). Task-cueing experiments usually consist of only two tasks and each task has an explicit task cue (Meiran, 2014). In each trial of Experiment 1, for example, an explicit frame cue was presented to indicate which task the participants should perform. In the following, tasks that stipulated the task cues and the task-switching rules were called *bivalent tasks*. Participants can apply the task-switching rules in order to switch between a filling and an orientation task. The target features that need to be categorized during performing the bivalent tasks were called *bivalent features*.

In the task-switching experiments with bivalent targets, researchers typically distinguished the difference between trials with congruent and incongruent target stimuli (e.g., Meiran and Kessler, 2008; Wendt and Kiesel, 2008; Schneider and Logan, 2009, 2014; Schneider, 2015, 2018; Li et al., 2019a). For example, in the present experiment, two bivalent tasks were the filling task and the orientation task. In the filling task, participants were required to press the left key if a rectangular bar was filled with black and the right key if the bar was unfilled. In the orientation task, participants were required to press the left key if the rectangular bar was vertical and the right key if the bar was horizontal. In this example, a filled vertical bar and an unfilled horizontal bar were congruent targets that lead to the same response in the filling and orientation task. In contrast, incongruent targets, filled horizontal targets and unfilled vertical targets, lead to different responses in each task. Participants typically have increased response times (RTs) and error rates (ERs) in trials with incongruent targets compared to congruent targets. These differences in RT and ER are known as response-congruency effects (Sudevan and Taylor, 1987). The response-congruency effects can emerge as a consequence of task rule-based feature categorization and the conflicting feature-response associations in incongruent trials, compared to the unique response in congruent trials (Schneider, 2015, 2018).

However, Li et al. (2019a) assigned each target an additional feature which was not related to bivalent tasks and bivalent features. For example, participants in their Experiment 2 were able to use these additional features to retrieve a response directly without knowing the color/orientation task-switching rules. Li et al. (2019a) found that participants still exhibit a reliable response-congruency effect, even without applying the task-switching rules. They proposed that although the task-switching rules were not introduced and the bivalent features were not necessary to the response selection process, participants could have formed bivalent feature-response associations automatically (Hommel, 1998, 2004, 2005) which resulted in the interference during response selection and the response-congruency effects.

According to the shielding function of categorization rules (Dreisbach and Haider, 2008, 2009; Dreisbach and Wenke, 2011; Dreisbach, 2012; Reisenauer and Dreisbach, 2013, 2014), we hypothesized that if participants could apply a simple categorization rule on the additional features, the participants may be able to focus on the relevant feature of the target and ignore the interference from the irrelevant bivalent features. Therefore, the response-congruency effect could be eliminated in trials in which the task-switching rules were not informed. To test this hypothesis, Experiment 1 assigned a *single digital numeral feature* to each target stimulus. We instructed participants to utilize the single digital numeral feature in Stage 1 and 2. Specifically, half of the participants were required to perform by numeral-response associations (association group). The other half of the participants were asked to apply a numeral categorization rule: if the numeral was odd, then they should press the left key; if the numeral was even, then they should press the right key (numeral-categorization group). In Stage 3, we displayed task cues and introduced the filling/orientation task-switching rules as in a typical task-cueing paradigm (Meiran, 2014).

We predicted that participants in the association group should show a significant response-congruency effect in all stages. In contrast, participants in the numeral-categorization group should only show significant response-congruency effects in Stage 3 when participants were informed about the task-switching rules. In Stage 1 and 2, the shielding function of the numeral-categorization rules should help participants ignore the interference from the bivalent features as those in Dreisbach and colleagues (Dreisbach et al., 2006, 2007; Dreisbach and Haider, 2008) and eliminate response-congruency effects. In terms of task-switching costs, according to previous results (Dreisbach et al., 2006, 2007; Dreisbach and Haider, 2008; Li et al., 2019a), all participants should show significant task-switching costs in Stage 3 where each participant was informed about the task cues and related task rules that required bivalent feature categorization. In Stage 1 and 2, participants should show no task-switching costs because they did not know the task rules yet.

### Methods

#### Participants

Forty-eight (34 females) university students from Fudan University voluntarily participated in this experiment (*M* = 24 years, *SD* = 3.94).

#### Research Ethics

Experiment 1 and 2 were reviewed and approved by the Fudan University, Department of Psychology Ethics Committee. Prior to the study, all participants were informed about the procedure of Experiment 1 and 2. All participants provided written, informed consent before taking part in Experiment 1 and 2.

#### Apparatus and Stimuli

Experiment 1 was programmed using PsyToolkit (Stoet, 2010, 2017). All stimuli were presented centrally on a 20-inch Lenovo computer monitor with a dark-green background (RGB: 128, 150, 0). A QWERTY keyboard was used to record participants’ responses with ±1 ms precision. Participants gave left and right responses by pressing “A” key and “L” key on the keyboard with their left and right index finger, respectively.

**FIGURE 1:**
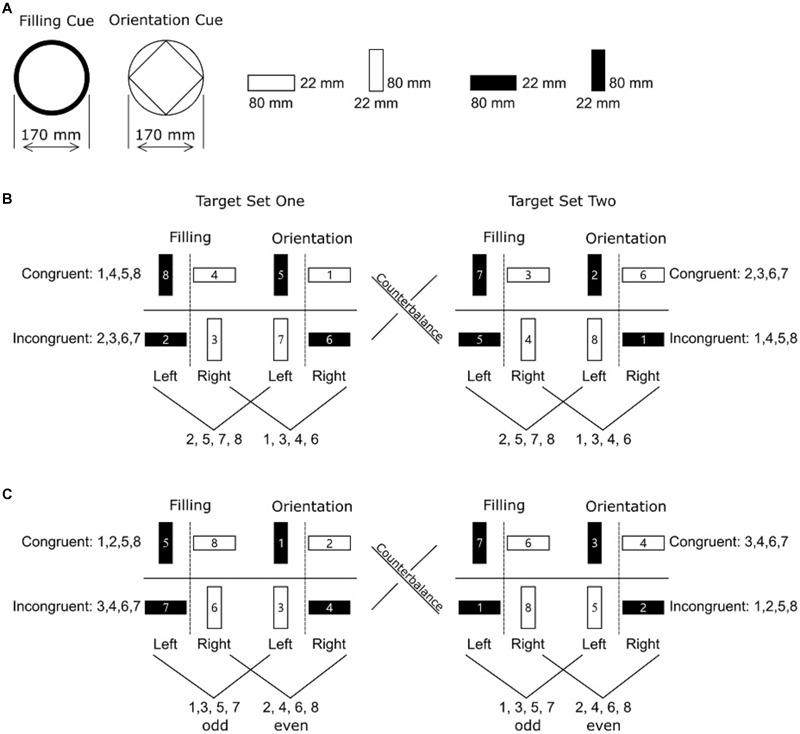
Illustration of cues, target stimuli, and response rules in Experiment 1. **(A)** Task cues and examples of bivalent features that participants needed to attend to in the filling task and orientation task. **(B)** Target stimuli and the response for the association group. Each target stimulus consisted of a single digital numeral presented centrally and a vertically or horizontally oriented rectangle bar either with or without filling. The digits for the congruent/incongruent target stimuli were counterbalanced between Target Set One and Target Set Two, giving a total of sixteen target stimuli for each participant. Digits 2, 5, 7, and 8 were always associated with the left key and digits 1, 3, 4, and 6 were always associated with the right key regardless of the bivalent features. **(C)** Target stimuli and the response for the numeral-categorization group. Participants should press the left key to respond to an odd number and the right key to respond to an even number, regardless of the bivalent features.

The target stimuli in the experiment consisted of four types of rectangle bars sharing feature fillings and orientations (i.e., unfilled horizontal bar, filled horizontal bar, unfilled vertical bar, and filled vertical bar). Each target stimulus had a single digital numeral (1, 2, 3, 4, 5, 6, 7, and 8) that was presented centrally and that was counterbalanced between bivalent tasks and conditions. Specifically,

(1) Each participant was given a total of 16 different target stimuli, with eight targets appearing only in the filling task and the other eight appearing only in the orientation task. Since each target stimulus was associated with only one possible response, participants could deduce the correct response without applying the filling/orientation task-switching rules.

(2) In order to avoid potential confounds between numeral-specific effects and the response congruency effects, every numeral appeared in both congruent and incongruent conditions. The digital numerals and their use in congruent/incongruent target stimuli were counterbalanced between the two target sets and two tasks.

(3) Because the numeral-categorization rule (i.e., odd-even categorization) can be easily deduced by participants, we gave each group of participants a different target-stimuli set. For the association group, rectangle bars and single digital numerals were arranged in such a way that participants could only perform by numeral-response associations (Figure 1B). For the numeral-categorization group, rectangle bars and single digital numerals were arranged in such a way that participants could apply the simple odd-even rule on the numerals to deduce the correct responses (Figure 1C).

#### Procedure

Participants were randomly assigned to one of the two groups: association group or numeral-categorization group (Figure 1). Participants were seated in front of a computer screen at a viewing distance of approximately 80 cm. On-screen instructions were displayed before each stage.

In Stage 1, the instructions stated that participants need to utilize the single digital numeral to deduce the correct response. Participants in the association group were instructed to memorize all target-response associations: the target with a digit 2, 5, 7, or 8 ⇒ left key; the target with a digit 1, 3, 4, or 6 ⇒ right key. Participants in the numeral-categorization rule group were instructed to apply a simple odd-even categorization rule: odd number ⇒ left key; even number ⇒ right key. In each trial, no task cues were presented. Since each target stimulus was associated with only one possible response, the participants should be able to deduce the correct response with the digital numerals alone. Here, we can classify task-repeat and task-switch trials without introducing task cues and bivalent tasks (i.e., filling and orientation tasks) because each bivalent task had a different set of target stimuli. If the targets in *trial n–1* and *trial n* belonged to the same task, *trial n* was a task-repeat trial, and otherwise *trial n* was a task-switch trial. Participants first carried out a training block of 32 trials followed by two experimental blocks with 128 trials each.

In each trial of Stage 2 and 3, the target stimulus and the task cue were presented simultaneously, with the target displaying inside a surround that served as a cue. In Stage 2, participants were instructed that the surrounding frames were meaningless and should be ignored when responding to or classifying the single-digit numerals. The reason for displaying the task cues in each trial of Stage 2 was to introduce cue-related distraction. This should make performance in Stage 2 and 3 more comparable. Participants completed two experimental blocks with 128 trials each.

In Stage 3, we introduced task cues and related bivalent tasks. Here, bivalent tasks were filling task and orientation task. In the filling task, the target stimulus that was filled with black was associated with a left-key press and the target stimulus that was unfilled was associated with a right-key press. In the orientation task, a vertical target stimulus was associated with a left-key press and a horizontal target stimulus was associated with a right-key press (Figure 1). Participants could either apply the task-switching rules or follow the strategies learned in the first two stages. Participants first carried out a training block with 32 trials followed by two experimental blocks with 128 trials each.

We controlled the target appearance, so that in all stages, the same target did not occur in consecutive trials. In each trial, if a correct response was made, the next trial would commence after a 300 ms inter-trial interval. If no response was made within 2.5 s, the text message “Timeout” was displayed. Incorrect responses were followed by the on-screen text message “Mistake.” Both feedbacks were visible for 3 s before the next trial started.

#### Data Analyses

In the following, we excluded error trials from RT analyses. The first trial of each block and trials immediately following an incorrect response were excluded from all analyses. If participants made an error in a previous trial, the subsequent trial could not be classified as a switch or repeat trial. We excluded all training trials from the analyses. In total, 4.76, 5.14, and 7.73% of the data were removed from the experimental blocks in Stage 1, Stage 2 and Stage 3, respectively. We used statistical software package R, version 3.4.2 (R Core Team, 2017) to analyze all data. Raw data are included in supplementary materials.

### Results

**FIGURE 2:**
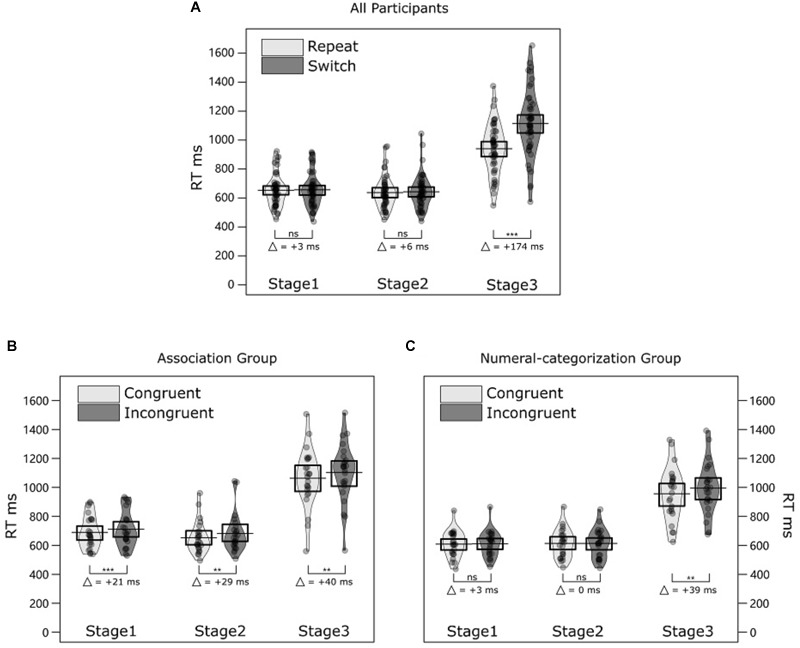
Results of Experiment 1. **(A)** The violin plots illustrate RT distributions for all 48 participants (24 participants from the association group and 24 participants from the numeral-categorization group) in repeat and switch trials and each stage (Stage 1, Stage 2, and Stage 3). Jittered dots represent individual average RTs. The black horizontal bar and the box represent the mean and 50% CI of the mean in each condition. **(B)** Violin plots illustrate RT distributions of congruent and incongruent trials for each stage in the association group. **(C)** Violin plots illustrate RT distributions of congruent and incongruent trials for each stage in the numeral-categorization group. ^∗∗∗^*p* < 0.001; ^∗∗^*p* < 0.05; and ns = non-significant.

Two four-way ANOVAs with mixed effects were conducted on mean RTs and ERs to compare different conditions. The three within-subjects factors were Trial transition (repeat, switch), Congruency (congruent, incongruent), and Stage (Stage 1, Stage 2, and Stage 3). The between-subject factor was Numeral group (association group, numeral-categorization group). The results of both analyses are summarized in Table 1 and illustrated in Figure 2. Mean values from each condition are listed in the Appendix.

**Table 1:** Experiment 1.

	Response time				Error rate			
Factor	*F*	*df*	*p*	$\eta_{p}^{2}$	*F*	*df*	*p*	$\eta_{p}^{2}$
NG	9.12	1, 46	0.004	0.166	3.74	1, 46	0.059	0.075
T	100.93	1, 46	<0.001	0.687	3.80	1, 46	0.057	0.076
C	34.19	1, 46	<0.001	0.426	15.06	1, 46	<0.001	0.247
S	152.96	2, 92	<0.001	0.769	1.01	2, 92	0.368	0.022
NG × T	<0.01	1,46	0.954	<0.001	0.53	1, 46	0.467	0.012
NG × C	4.27	2, 92	0.044	0.085	1.20	2, 92	0.279	0.025
NG × S	0.58	1, 46	0.557	0.013	0.04	1, 46	0.959	0.001
T × C	2.82	2, 92	0.100	0.058	0.03	2, 92	0.853	<0.001
T × S	90.04	2, 92	<0.001	0.662	<0.001	2,92	0.998	<0.001
C × S	10.46	1, 46	<0.001	0.185	3.01	1, 46	0.054	0.061
NG × T × C	0.01	2, 92	0.934	<0.001	0.44	2, 92	0.057	0.010
NG × T × S	0.08	2, 92	0.921	0.002	0.73	2, 92	0.483	0.016
NG × C × S	2.87	2, 92	0.061	0.059	0.11	2, 92	0.896	0.002
T × C × S	0.21	2, 92	0.810	0.005	0.94	2, 92	0.393	0.020
NG × T × C × S	0.69	2, 92	0.503	0.012	0.39	2, 92	0.677	0.008

*Results of ANOVAs on mean RTs and ERs, with Trial transition (repeat, switch), Congruency (congruent, incongruent), and Stage (Stage1, Stage2, and Stage3) as within-subjects factors, and Numeral group (association group, numeral-categorization group) as the between-subjects factor. NG = Numeral group; T = Trial transition; C = Congruency; and S = Stage.*

Response times were longer and ERs higher in the incongruent trials (786 ms, 6.03%) compared to the congruent trials (764 ms, 3.60%). The RTs were longer in switch trials (805 ms) compared to repeat trials (744 ms). Participants in the association group responded slower (814 ms) compared to participants in the numeral-categorization group (730 ms). In the following, *post hoc* pairwise comparisons were always adjusted for multiple comparisons after Holm (1979). When comparing RT differences between stages, the RT difference between Stage 1 (648 ms) and Stage 2 (640 ms) was not significant, *t*(47) = 0.58, *p* = 0.577. The difference between Stage 1 and Stage 3 (1029 ms) was significant, *t*(47) = -13.61, *p* < 0.001; as was the difference between Stage 2 and 3, *t*(47) = -12.92, *p* < 0.001.

There was a significant interaction between Trial transition and Stage (T × S) in the RT analysis. A *post hoc* comparison indicated that RT switching costs were not significant in Stage 1 (switch – repeat = +3 ms), *t*(47) = 1.10, *p* = 0.275; and Stage 2 (switch – repeat = +6 ms), *t*(47) = 1.76, *p* = 0.168; but statistically significant in Stage 3 (switch – repeat = +174 ms), *t*(47) = 9.99, *p* < 0.001.

For RT, the interaction between Congruency and Stage was significant. The interaction between Congruency and Numeral group was also significant. The three-way interaction (Numeral group × Congruency × Stage) was approaching significance. In order to better interpret these interactions, we analyzed the RT congruency effect for each stage and each numeral group. The results show that in the association group, response-congruency effects were significant in all stages: Stage 1 (incongruent – congruent = +21 ms), *t*(23) = 3.20, *p* = 0.012; Stage 2 (incongruent – congruent = +29 ms), *t*(23) = 4.41, *p* < 0.001; and Stage 3 (incongruent – congruent = +40 ms), *t*(23) = 4.08, *p* = 0.002. In contrast, in the numeral-categorization group, response-congruency effects were significant in Stage 3 (incongruent – congruent = +39 ms), *t*(23) = 3.41, *p* = 0.008, but not in Stage 1 (incongruent – congruent = +3 ms), *t*(23) = 0.65, *p* = 0.524 or Stage 2 (incongruent – congruent = 0 ms), *t*(23) < 0.001, *p* > 0.999 (Figure 2). No other effect reached statistical significance.

#### Congruency Effects and Task Type

In two additional three-way ANOVAs we investigated whether there were differences in RT and ER congruency effects for participants in the association group between filling and orientation tasks and in Stage 1 and 2. The three within-subject factors were Congruency (congruent, incongruent), Stage (Stage 1, Stage 2) and Task (filling, orientation).

For RT, the main effect of Congruency was significant, *F*(1, 23) = 18.88, *p* < 0.001, $\eta_{p}^{2}$ = 0.451. The interaction between Congruency and Task was significant, *F*(1, 23) = 7.47, *p* = 0.012, $\eta_{p}^{2}$ = 0.243. A *post hoc* pairwise comparison showed that in Stage 1, the congruency effects were only significant in the orientation task (incongruent – congruent = +42 ms), *t*(23) = 4.46, *p* < 0.001; but not in the filling task (incongruent – congruent = +2 ms), *t*(23) = 0.16, *p* = 0.872. However, in Stage 2, the congruency effects were significant in both the orientation task (incongruent – congruent = +39 ms), *t*(23) = 4.48, *p* < 0.001 and filling task (incongruent – congruent = +20 ms), *t*(23) = 2.13, *p* = 0.043. The magnitude of RT congruency effects were not significantly different between the two tasks, *t*(23) = 1.55, *p* = 0.13.

For ER, the main effect of Congruency was significant (incongruent – congruent = +0.94%), *F*(1, 23) = 10.75, *p* = 0.003, $\eta_{p}^{2}$ = 0.319. The interaction between Task and Stage was significant, *F*(1, 23) = 10.15, *p* = 0.004, $\eta_{p}^{2}$ = 0.306. In Stage 1, the ER difference between the filling (2.51%) and orientation task (3.89%) was significant, *t*(23) = 2.88, *p* = 0.017. In Stage 2, the difference between filling (2.88%) and orientation task (3.96%) was not significant, *t*(23) = 0.36, *p* = 0.719. No other results reached statistical significance.

#### Verbal Report

All participants reported that they performed filling and orientation tasks in Stage 3. In Stage 1 and 2, participants in the association group reported that they applied numeral-response associations (for example, “2” ⇒ left key) and participants in the alternative rule group reported that they applied the numeral-categorization rule as instructed (i.e., odd numeral ⇒ left key; even numeral ⇒ right key).

### Discussion

In Stage 1 and 2, we found no task-switching costs when participants were instructed to utilize either the numeral-response associations or numeral-categorization rules to deduce correct responses. In Stage 3, we introduced task cues and bivalent tasks (i.e., filling and orientation task), and participants began to show task-switching costs. These results replicated the previous findings that task-switching costs only appear when participants applied task-switching rules (Dreisbach et al., 2006, 2007; Dreisbach and Haider, 2008; Li et al., 2019a,b):

Participants in both the association group and numeral-categorization group indicated reliable response-congruency effects in Stage 3 where task cues and bivalent tasks were introduced. The response-congruency effects can be due to rule-based feature categorization and conflicting feature-response selection in incongruent trials (Schneider, 2015, 2018). However, we found that, in Stage 1 and 2, participants in the association group had a reliable response-congruency effect, even though the bivalent tasks were not informed, whereas participants in the numeral-categorization group showed no response-congruency effects.

In Stage 1 and 2, all participants reported that they utilized the numeral features to infer a correct response. Nevertheless, participants in the association group may still have perceived the bivalent features and formed bivalent feature-response associations automatically (Hommel, 1998, 2004, 2005) and therefore produced response-congruency effects (Li et al., 2019a).

In contrast, participants who applied the numeral-categorization rules indicated no response-congruency effects in Stage 1 and 2. The numeral-categorization rule helped participants to allocate attention toward the numeral features of the target (i.e., parity), and helped participants to bypass the currently irrelevant information such as fillings and orientations. The absence of the response-congruency effects in Stage 1 and 2 of the numeral-categorization group was consistent with the shielding function of the categorization rules in previous studies (Dreisbach and Haider, 2008, 2009; Reisenauer and Dreisbach, 2013, 2014; Bogon et al., 2017).

#### Response-Congruency Effects in Different Tasks

An alternative interpretation of the response-congruency effects in Stage 1 and 2 is that participants in the association group may utilize one of the bivalent features (filling or orientation) to predict a response. For example, participants could associate the left key with filled bars and the right key with unfilled bars. This approach would predict the correct response in 75% of all trials (Figure 1), but was invalid and might delay the response in the remaining 25% of the trials with incongruent target stimuli. In this example, because the remaining 25% were incongruent trials in the orientation tasks, this may explain the response-congruency effects in Stage 1 and 2 but only in the orientation task trials. Similarly, if participants learned to associate the left key with vertical bars and the right key with horizontal bars, the response-congruency effects should only be significant in the filling task but not in the orientation task.

The results in Stage 1 support the interpretation that participants might have learned to associate the left key with filled bars and right key with unfilled bars because they only showed response-congruency effects in the orientation task (+42 ms) but not in the filling task (+2 ms). However, there was little evidence that participants applied a similar approach in Stage 2 because the response-congruency effects were significant and equivalent in both tasks.

A “feature difficulty” account may provide a simpler explanation for the response-congruency effects in different tasks. Previous studies showed that response-congruency effects were larger in one task when the other task features were easier to detect (Meiran and Kessler, 2008; Schneider, 2018; Li et al., 2019a). Perhaps the orientation features are harder to detect compared to filling features (Li et al., 2019a). Although there were no RT differences between the filling and orientation tasks, we found that participants had higher ERs in the orientation task compared to the filling task in Stage 1 while the ERs were equal between the two tasks in Stage 2. These findings suggest that participants may perceive the filling task feature as easier in Stage 1 and therefore showed larger response-congruency effects in the orientation task trials. However, after some practice, in Stage 2, both the orientation and filling features appear to have been learned equally well by participants, and the congruency effects were significant in both tasks and equal between the two tasks.

## Experiment 2

In Experiment 2, we sought to explore the limitation of the shielding function of the categorization rules. We suspected that when the distracting features were strong, the shielding function of the categorization rules should not be able to keep the interference away from the focus of attention. We used a similar task-switching paradigm as in Experiment 1. In order to examine the limitation of the shielding function, in Experiment 2 we applied new bivalent features that might cause larger interference during response selections and produce larger response-congruency effects.

**FIGURE 3:**
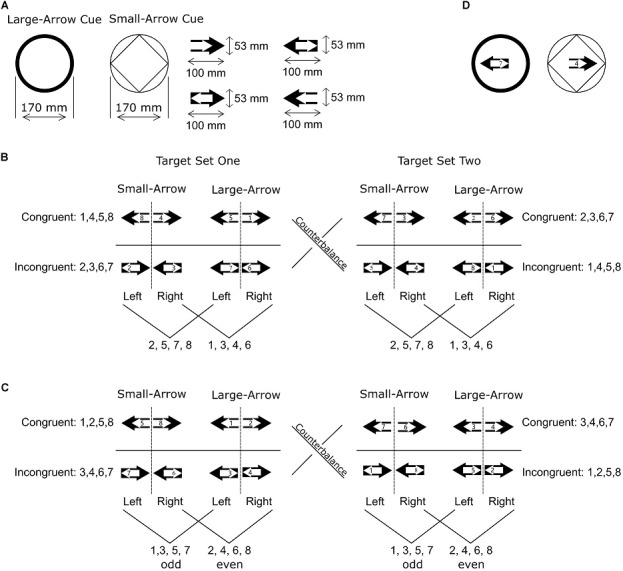
Illustration of cues, target stimuli, and the corresponding responses in Experiment 2. **(A)** Task cues and examples of the arrow features of target stimuli. **(B)** Target stimuli for the association group. Each target stimulus consisted of two arrows. Digits 2, 5, 7, and 8 were always associated with the left key and digits 1, 3, 4, and 6 were always associated with the right key. **(C)** Target stimuli for the numeral-categorization group. Participants should press the left key to respond to an odd number and the right key to respond to an even number. **(D)** An example of cue-target combination. A single digital numeral was always presented in the center.

Response-congruency effects are larger when “categorization” of the irrelevant task dimensions is easier (Schneider, 2018), or when the irrelevant task feature is easier to detect (Li et al., 2019a). In order to make task features easier to detect and trigger larger response-congruency effects, each target in Experiment 2 contained a large filled arrow and a small unfilled arrow as bivalent features (Figure 3). Arrows have been used in various cognitive experiments to create interference, such as an arrow version of the Eriksen flanker task (Fan et al., 2002), and global/local interference task (Weinbach and Henik, 2014). In these studies, arrows that pointed in the same direction (both pointing toward left or right) produced no interference during response selections whereas arrows that pointed to the opposite directions led to the reduced efficiency of interference control and produced large response-congruency effects.

As in Experiment 1, we assigned a single digital numeral to each target stimulus. In Stage 1 and Stage 2, participants were required to utilize the numerals to infer correct responses so that the bivalent features (two arrows) were irrelevant to response selections. Again the combination of arrows and numerals were different between two group of participants, so that one group of participants could only perform by numeral-response associations and the other group could only apply the numeral-categorization rules. In Stage 3, we informed participants as to task cues and bivalent tasks to respond in the direction indicated by an arrow.

In Experiment 2, we predicted that the arrow feature of the target stimuli would be easily detected by participants. That is, that the arrow feature would produce strong interference exceeding the limitation of the shielding function of task rules. Hence, we predicted that participants in both the association group and numeral-categorization group should show significant response-congruency effects in all stages.

In line with Experiment 1 and previous results (Dreisbach and Haider, 2008; Li et al., 2019a), we further predicted that all participants should show significant task-switching costs in Stage 3 when the bivalent tasks were introduced. By contrast, in Stage 1 and 2, participants should show no task-switching costs because they do not know the bivalent tasks yet.

### Methods

#### Participants

A new sample of fifty-two university students (38 females) from Fudan University voluntarily participated in this experiment (*M* = 26 years, *SD* = 4.24).

#### Apparatus and Stimuli

Apparatus was identical to Experiment 1, but new target stimuli were applied. In Experiment 2, each target stimulus included a large and a small arrow, and the arrows pointed in the left and right directions, leading to four types of arrow combinations: (1) large left arrow + small left arrow; (2) large right arrow + small right arrow; (3) large left arrow + small right arrow; (4) large right arrow + small left arrow. A single digital numeral was assigned to each target stimulus in a way that was identical to Experiment 1 (Figure 3B,C). The size of each target stimulus was 53 × 100 mm. In Stage 2 and 3, task cues were displayed, while participants were informed of the meaning of the task cues only in Stage 3. The two task cues were different surrounds with a diameter of 170 mm. Cues and target stimuli were presented on a dark-green background (RGB: 128, 150, 0).

#### Procedure

Instructions for Stage 1 and Stage 2 were identical to Experiment 1. However, in Stage 3, we introduced task cues, bivalent tasks and relevant task rules. Here, bivalent tasks were large-arrow task and small-arrow task. A circle cue signaled to determine whether the large arrow pointed to either the left or the right (left ⇒ press the left key; right ⇒ press the right key). A circle with inscribed square signaled to determine whether the small arrow pointed to either the left or right side (left ⇒ press the left key; right ⇒ press the right key; Figure 3). In Stage 1, participants first carried out a training block of 32 trials followed by two experimental blocks with 128 trials each. There was no training block in Stage 2: participants carried out two experimental blocks of 128 trials each. In Stage 3, participants first carried out a training block of 32 trials followed by two experimental blocks with 128 trials each.

#### Data Analyses

We used the same trial exclusion criteria as in Experiment 1. As a result, 5.34, 5.56, and 5.89% of the data had to be removed from the experimental blocks in Stage 1, Stage 2, and Stage 3, respectively.

### Results

**FIGURE 4:**
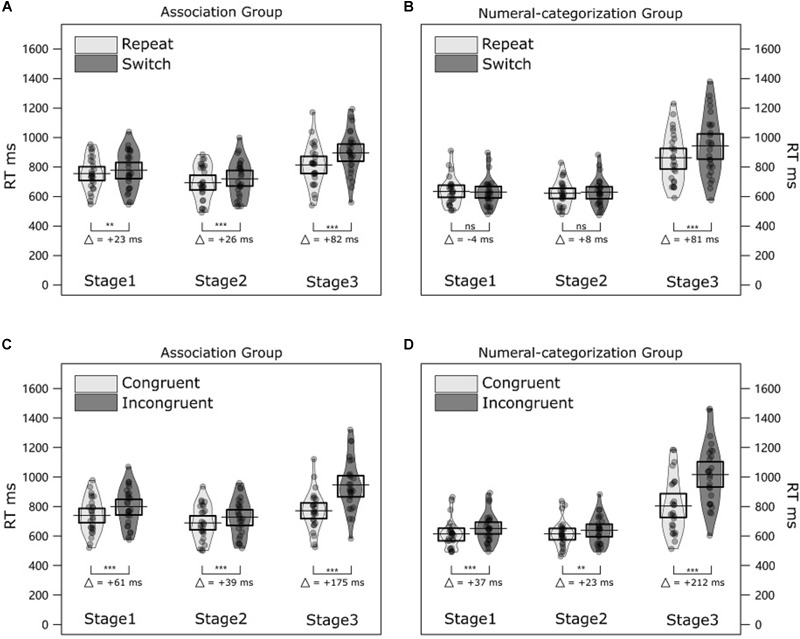
Results of Experiment 2. **(A)** The violin plots illustrate RT distributions for participants in the numeral-response association group for repeat and switch trials across stages (Stage 1, Stage 2, and Stage 3). Jittered dots represent individual average RTs. The black horizontal bar and the box represent the mean and 50% CI of the mean in each condition. **(B)** The violin plots illustrate RT distributions for participants in the numeral-categorization group for repeat and switch trials across stage. **(C)** The violin plots illustrate RT distributions for participants in the association group for the congruent and incongruent trials and each stage. **(D)** The violin plots illustrate RT distributions for participants in the numeral-categorization group for the congruent and incongruent trials across stages. ^∗∗∗^*p* < 0.001; ^∗∗^*p* < 0.05; and ns = non-significant.

Two four-way ANOVAs with mixed effects were conducted on mean RTs and ERs to compare different conditions. Trial transition (repeat, switch), Congruency (congruent, incongruent) and Stage (Stage 1, Stage 2, and Stage 3) were within-subject factors and Numeral group (association group, numeral-categorization group) was a between-subject factor. The results of both analyses are summarized in Table 2 and illustrated in Figure 4. Mean values of each condition are listed in the Appendix.

**Table 2:** Experiment 2.

	RT				ER			
Factor	*F*	*df*	*p*	$\eta_{p}^{2}$	*F*	*df*	*p*	$\eta_{p}^{2}$
NG	3.38	1, 50	0.071	0.063	2.35	1, 50	0.131	0.045
T	92.19	1, 50	<0.001	0.648	0.16	1, 50	0.685	0.003
C	169.97	1, 50	<0.001	0.773	86.01	1, 50	<0.001	0.632
S	85.19	2, 100	<0.001	0.630	1.52	2, 100	0.223	0.030
NG × T	4.75	1, 50	0.034	0.087	0.09	1, 50	0.764	0.002
NG × C	<0.01	1,50	0.925	<0.001	0.71	1, 50	0.403	0.014
NG × S	14.22	2, 100	<0.001	0.222	0.02	2, 100	0.983	<0.001
T × C	0.59	1, 50	0.448	0.012	0.59	1, 50	0.444	0.012
T × S	55.82	2, 100	<0.001	0.528	6.21	2, 100	<0.001	0.111
C × S	69.70	2, 100	<0.001	0.582	0.19	2, 100	0.823	0.004
NG × T × C	<0.01	1,50	0.969	<0.001	0.07	1, 50	0.794	0.001
NG × T × S	1.13	2, 100	0.326	0.022	0.96	2, 100	0.387	0.019
NG × C × S	2.15	2, 100	0.122	0.041	3.02	2, 100	0.053	0.057
T × C × S	0.06	2, 100	0.944	0.001	2.99	2, 100	0.055	0.057
NG × T × C × S	1.22	2, 100	0.299	0.024	0.55	2, 100	0.580	0.011

*Results of ANOVAs on mean RTs and ERs, using Trial transition (repeat, switch), Congruency (congruent, incongruent), and Stage (Stage1, Stage2, and Stage3) as within-subjects factors, and Numeral group (association group, numeral-categorization group) as the between-subjects factor. NG = numeral group; T = trial transition; C = congruency; and S = stage.*

Response times were longer and ERs higher in the incongruent trials task (797 ms, 5.47%) compared to the congruent trial (705 ms, 1.76%). RTs were longer in switch trials (767 ms) compared to repeat trials (731 ms). Participants in the association group responded more slowly (814 ms) compared to participants in the numeral-categorization group (730 ms). In the following, *post hoc* pairwise comparisons were always adjusted for multiple comparisons after Holm (1979). For the factor of Stage in the RT analysis, we found that participants had shorter RTs in Stage 2 (667 ms) than in Stage 1 (694 ms); *t*(51) = -2.52, *p* = 0.014. Participants had longer RT in Stage 3 (881 ms) than in Stage 1, *t*(51) = 7.41, *p* < 0.001 and Stage 2, *t*(51) = 10.16, *p* < 0.001.

#### Congruency Effects

Congruency significantly interacted with Stage in RT. A *post hoc* comparison indicated that the congruency effects were significant in all stages: Stage 1 (incongruent – congruent = +49 ms), *t*(51) = 7.27, *p* < 0.001; Stage2 (incongruent – congruent = +31 ms), *t*(51) = 6.12, *p* < 0.001; and Stage 3 (incongruent – congruent = +194 ms), *t*(51) = 10.90, *p* < 0.001. However, the magnitude of the congruency effects varied across stages. Congruency effects were larger in Stage 1 than in Stage 2, *t*(51) = 2.40, *p* = 0.020. Congruency effects were larger in Stage 3 than in Stage 1, *t*(51) = 7.71, *p* < 0.001. Congruency effects were larger in Stage3 than in Stage 1, *t*(51) = 9.04, *p* < 0.001.

In order to examine whether the congruency effects were significant in both groups and all stages, we compared individual mean RTs and ERs in congruent trials with incongruent trials in each stage and for each group of participants. For RT analysis, the corresponding paired *t*-tests showed that in the association group, the congruency effects were significant in all stages: Stage 1 (incongruent – congruent = +61 ms), *t*(25) = 5.13, *p* < 0.001; Stage 2 (incongruent – congruent = +39 ms), *t*(25) = 6.07, *p* < 0.001; and Stage 3 (incongruent – congruent = +175 ms), *t*(25) = 7.31, *p* < 0.001. In the numeral-categorization group, congruency effects were also significant in all stages: Stage 1 (incongruent – congruent = +37 ms), *t*(25) = 6.46, *p* < 0.001; Stage 2 (incongruent – congruent = +23 ms), *t*(25) = 3.03, *p* = 0.006; and Stage 3 (incongruent – congruent = +212 ms), *t*(25) = 8.06, *p* < 0.001. There was no difference between the RT congruency effects in the association group and numeral-categorization group in any stages (all *p* > 0.05).

For ER, the corresponding paired *t* tests showed that participants in the association group had significant congruency effects in all stages: Stage 1 (incongruent – congruent = +3.37%), *t*(25) = 6.32, *p* < 0.001; Stage 2 (incongruent – congruent = +2.22%), *t*(25) = 4.94, *p* < 0.001; and Stage 3 (incongruent – congruent = +4.84%), *t*(25) = 6.52, *p* < 0.001. Participants in the numeral-categorization rule group had also significant congruency effects in all stages: Stage 1 (incongruent – congruent = +2.79%), *t*(25) = 4.94, *p* < 0.001. Stage 2 (incongruent – congruent = +3.43%), *t*(25) = 5.53, *p* < 0.001. Stage 3 (incongruent – congruent = +5.65%), *t*(25) = 4.79, *p* < 0.001. There was no difference between the ER congruency effects in association group and numeral-categorization group in any stages (all *p* > 0.05).

#### Trial Transition

For the RT analysis, Trial transition significantly interacted with Numeral group. Trial transition significantly interacted with Stage, and Numeral group significantly interacted with Stage. In order to better interpret these two-way interactions, we analyzed the RT switching costs for each stage and each numeral groups. The corresponding paired *t*-tests showed that, in the association group, switching costs were significant in all stages: Stage 1 (switch – repeat = +23 ms), *t*(25) = 2.89, *p* = 0.008; Stage 2 (switch – repeat = +26 ms), *t*(25) = 3.69, *p* = 0.002; and Stage 3 (switch – repeat = +82 ms), *t*(25) = 7.01, *p* < 0.001. In contrast, in the numeral-categorization group, switching costs were significant only in Stage 3 (switch – repeat = +81 ms), *t*(25) = 7.48, *p* < 0.001, but not in Stage 1 (switch – repeat = -4 ms), *t*(25) = 0.52, *p* = 0.606, and Stage 2 (switch – repeat = +8 ms), *t*(25) = 1.45, *p* = 0.316 (Figure 4).

For the ER analysis, Trial transition significantly interacted with Stages. A *post hoc* comparison indicated that switching costs were significant in Stage 1 (switch – repeat = +0.86%), *t*(51) = 2.78, *p* = 0.015 and Stage 3 (switch – repeat = +2.3%), *t*(51) = 4.71, *p* < 0.001. In Stage 2, switching costs were not significant, *t*(51) = 0.80, *p* = 0.430.

#### Congruency Effects and Task Type

In two additional three-way ANOVAs, we investigated whether there were differences in RT and ER congruency effects for all participants between small-arrow and large-arrow tasks and in Stage 1 and 2. The three within-subject factors were Congruency (congruent, incongruent), Stage (Stage 1, Stage 2), and Task (large arrow, small arrow).

For RT, all three main effects were significant: Task, *F*(1, 51) = 4.74, *p* = 0.034, $\eta_{p}^{2}$ = 0.085; Congruency, *F*(1, 51) = 72.98, *p* < 0.001, $\eta_{p}^{2}$ = 0.589; and Stage, *F*(1, 51) = 6.92, *p* = 0.011, $\eta_{p}^{2}$ = 0.119. The interaction between Task and Stage was significant, *F*(1, 51) = 4.07, *p* = 0.049, $\eta_{p}^{2}$ = 0.074. A *post hoc* pairwise comparison showed that in Stage 1, the RT difference between the small-arrow task (710 ms) and the large-arrow task (693 ms) was significant, *t*(51) = 2.89, *p* = 0.011. However, in Stage 2, the RT difference between the small-arrow task (675 ms) and the large-arrow task (671 ms) was not significant, *t*(51) = 0.63, *p* = 0.533. The interaction between Congruency and Stage was significant, *F*(1, 51) = 5.31, *p* = 0.025, $\eta_{p}^{2}$ = 0.094. *Post hoc* comparison suggests that the congruency effects were larger in Stage 1 (incongruent – congruent = +48 ms) than in Stage 2 (incongruent – congruent = +32 ms), *t*(51) = 2.26, *p* = 0.028.

The interaction between Task and Congruency was not significant, *F*(1, 51) = 0.78, *p* = 0.379. The interaction between Task, Congruency, and Stage was also not significant, *F*(1, 51) = 1.02, *p* = 0.315. Planned comparisons suggest that congruency effects were significant in both tasks in Stage 1 and 2. In Stage 1, congruency effects were +58 ms in the small-arrow task, *t*(51) = 5.74, *p* < 0.001; and +40 ms in the large-arrow task, *t*(51) = 4.10, *p* < 0.001. In Stage 2, congruency effects were +35 ms in the small-arrow task, *t*(51) = 4.31, *p* < 0.001; and +35 ms in the large-arrow task, *t*(51) = 3.31, *p* = 0.002. The magnitude of Congruency effects was equivalent in both tasks in Stage 1 and 2 (all *p* > 0.05).

For ER, the main effect of Congruency was significant (incongruent – congruent = +2.95%), *F*(1, 51) = 90.76, *p* < 0.001, $\eta_{p}^{2}$ = 0.640. No other results reached statistical significance.

#### Verbal Report

All participants reported that they performed tasks of discriminating between the direction of large arrows and small arrows in Stage 3. In Stage 1 and 2, participants in the association group reported that they performed by numeral-response associations (for example, “2” ⇒ left key) and participants in the alternative rule group reported that they applied the numeral-categorization rule as informed (i.e., odd numeral ⇒ left key; even numeral ⇒ right).

### Discussion

In Experiment 2, participants from both the association group and numeral-categorization group showed significant response-congruency effects in all stages. In Stage 1 and 2, participants utilized single digital numerals to infer correct responses. This means that two arrows were irrelevant to the response selection and were considered as distractors. According to the results of Experiment 1 and the shielding function of the categorization rules in previous studies (Dreisbach and Haider, 2008, 2009), participants in the numeral-categorization group should have been able to bypass the interference from the two arrows and eliminate the response-congruency effects. However, we found that participants who applied numeral-categorization rules showed significant response-congruency effects similar to the association group. In other words, two arrows produced strong interference that reduced the efficiency of the shielding function of categorization rules.

There were differences between the two groups of participants in terms of RT task-switching costs. Participants in the numeral-categorization group showed significant task-switching costs only in Stage 3 but not in Stage 1 and 2. In contrast, surprisingly, we found that participants in the association group had task-switching costs in all three stages. We introduced task cues and bivalent tasks only in Stage 3, whereas in Stage 1 and Stage 2 participants performed the experiment using numeral-response associations as they reported in the post-experiment verbal report. In previous task-switching studies, researchers suggested that sometimes participants were able to deduce the task rules and use the rules implicitly (Meier et al., 2016; Li et al., 2019b). However, there were no task cues in Stage 1 of Experiment 2; therefore, suggesting that applying arrow rules implicitly might be impractical. We will discuss these unexpected task-switching costs in the General Discussion.

#### Response-Congruency Effects in Different Tasks

In Stage 1 and 2, if participants always followed the large arrow, this would predict the correct response in 75% of all trials (Figure 3). Since the remaining 25% were incongruent trials in the small-arrow tasks, this may explain the response-congruency effects in Stage 1 and 2 but only in the small-arrow trials. Similarly, if participants learned to only follow the small arrow, the response-congruency effects should only be significant in the small-arrow task but not in the large-arrow task. Nevertheless, in Stage 1 and 2, response-congruency effects were significant in both large-arrow and small-arrow tasks and equivalent between the two tasks. These alternative approaches was not compatible with the results of Experiment 2.

## General Discussion

In Experiment 1, by comparing the response-congruency effects between the numeral-association group and numeral-categorization group, we replicated the shielding function of categorization rules. In Stage 1 and 2, when participants performed by numeral-response associations, they received interference from the irrelevant feature-response associations and showed reliable response-congruency effects. In contrast, participants who learned to apply the numeral-categorization rules eliminated the response-congruency effects because the shielding function of the categorization rules prevented participants from receiving interference from the irrelevant features. However, in Experiment 2, when irrelevant features produced large interference, the shielding function of categorization rules failed and participants showed significant congruency effects in all stages. We suggested that the shielding function of categorization rules had limitations in terms of blocking irrelevant features.

Recent studies proposed that the shielding function of categorization rules might reflect advantages in the processing of relevant features rather than suppressing the irrelevant features (Dreisbach and Wenke, 2011; Reisenauer and Dreisbach, 2013, 2014). This is in line with the relative-speed-of-processing hypothesis proposed by Schneider (2018) that suggested that distracting features can interfere with responses only when participants start processing them before they complete the response selection process. Similarly, it is possible that in the Stage 1 and 2 of Experiment 1 participants who applied the numeral-categorization rules processed numeral features (odd-even) more quickly. Thus, response selection processes related to numeral-categorizations may have already been completed before participants processed other features (e.g., filling and orientation). As a consequence, there were little or no conflicts during response selection, and the response-congruency effects disappeared.

In Stage 1 and 2 of Experiment 2, the bivalent features were two overlapping arrows. Arrows are important distractors that can create strong interference in different types of experiments (Fan et al., 2002; Weinbach and Henik, 2014). Arrow signals have also been widely used in daily life. Therefore, we speculate that processing of arrow signs may be prioritized and fast. As a result, although the numeral categorization rules specifically should have guided participants’ attention to an odd-even feature of the target, participants still processed the arrows quickly and received interference during response selection in the incongruent trials. It has long been demonstrated that participants can prioritize some features better than others by default. For example, in the well-known Stroop task where participants are required to name the ink color of a color word, word processing is faster than ink-color processing because more attention is allocated to recognize the color of the word compared to the meaning of the word (c.f., MacLeod, 1991).

### Unexpected Task-Switching Costs

Previous studies showed that task-switching costs only exist when participants were informed about bivalent features and task-switching rules (Dreisbach and Haider, 2008; Li et al., 2019a,b; but see Forrest et al., 2014). However, inconsistent with previous results, participants in the association group of Experiment 2 showed task-switching costs in Stage 1 and 2 even without introducing the bivalent features and related task rules. Task-switching costs indicate an extra cognitive effort on reconfiguring the task set or resolving proactive interference in task-switching trials (e.g., Kiesel et al., 2010; Vandierendonck et al., 2010). Without introducing or knowing bivalent features as well as related task-switching rules, participants should not have shown any task-switching costs in Stage 1 and 2 of Experiment 2.

One possible explanation for the unexpected task-switching costs in Experiment 2 could be that, even without knowing bivalent tasks, participants in the association group might still have received proactive interference from the preceding bivalent feature-response associations which produced task-switching costs. Previous studies have proposed that proactive interference can be produced by bivalent features (Woodward et al., 2003). Here, we further suggest that bivalent features may produce proactive interference automatically without applying corresponding task rules. Taking a small-arrow task as an example: In trials of the small-arrow task, the small arrow always pointed to the correct response but the large arrow sometimes pointed to the wrong response key and need to be inhibited. If the following trial is a trial of a large-arrow task in which the large arrow always points to the correct response, the large arrow needs to be reactivated due to the previous suppression. The reactivation process may delay the response in the task-switching trials and may therefore be the cause of the resultant task-switching costs.

### Response-Congruency Effect and Task-Switching Costs

Both response-congruency effects and task-switching costs can be produced by the interference of automatically formed bivalent feature-response associations, but with different timing. The former can be caused by the immediate interference between two concurrent feature-response associations: The interference is elicited from the external environment. The latter can be produced by the proactive interference from the feature-response associations in the preceding trials: The interference is elicited from information in memory. Prior evidence suggests that two types of interference may reflect different cognitive processes (Friedman and Miyake, 2004; but see Pettigrew and Martin, 2014).

We suspect that, normally, without participants knowing bivalent features and related task rules, the proactive interference that is produced by automatically formed bivalent feature-response associations may be too weak to produce task-switching costs. Thus, in Li et al. (2019a), as well as in Experiment 1 of the present study, the task-switching costs were undetectable until bivalent features and task-switching rules were introduced. However, the bivalent features were two arrows in Experiment 2. As participants might prioritize the arrow features over other features, the proactive interference produced by arrow-response associations is strong enough to produce task-switching costs even without knowing the arrow-related bivalent task rules.

Moreover, it appears that, in Experiment 2, proactive interference that produces task-switching costs may be weaker than the immediate interference that produces response-congruency effects. Without knowing rules related to arrow tasks, these cases of automatically formed proactive interference were not strong enough to penetrate the shielding function of categorization rules. As demonstrated in Experiment 2, when participants applied the numeral-categorization rule, there were no task-switching costs but the response-congruency effects remained. Nevertheless, it remains unclear why the proactive interference from working memory is weaker than the immediate interference that is caused by the conflicts during response selection in the present trial. Further investigations may be necessary to clarify the difference.

### Strategies in Task-Switching

In line with Li et al. (2019a), we found that even though some participants learned to perform tasks by using the numeral features associatively in Stage 1 and 2, in Stage 3 these participants preferred to use bivalent features to retrieve a response, indicating task-switching costs and on average yielding slower responses. Note that we introduced task cues, bivalent features, and related task rules in Stage 3 without demanding that participants should apply these rules. According to previous studies, participants might prefer strategies that prioritize goal-related information and reduce task uncertainty (Mackie et al., 2013; Cooper et al., 2015; Li et al., 2019a).

However, half of the participants learned simple numeral-categorization rules in the two experiments of the present study (Stage 1 and 2), and yet they still applied task-switching rules and showed task-switching costs as soon as the bivalent features and the rules were introduced (Stage 3). Therefore, a simpler explanation is that participants merely followed the instructions in every stage.

### Limitation and Future Direction

In Experiments 1 and 2, in order to make sure participants in the association group could not apply the odd-even categorization rule on numerals, the target stimuli in the association group were different from those in the categorization-rule group. We suggest that future study could use language barrier to control the use of odd-even categorization rules in the experiment with numeral targets (Li et al., 2019b). For example, if the Arabic numerals are replaced with Chinese numeral characters, only participants who can read Chinese numerals are able to apply the odd-even categorizations whereas those who do not know and cannot read Chinese should only utilize those numeral characters associatively (Li et al., 2019b).

Reisenauer and Dreisbach (2013, 2014) showed that when interference from the distractor pictures and the word categorization rules were semantically related to each other—that is, when the interference can also be categorized along the categorization rule—the shielding function of the word categorization rule cannot prevent the interference. Perhaps, in such cases, the focus of attention is narrowed and allocated to both the interference and the relevant features, so that the shielding function failed. To avoid this problem, in the present study, we demonstrated the shielding function by using target stimuli with the irrelevant features and the categorization rule that were semantically unrelated. In both Experiment 1 and 2, the odd-even categorization rule cannot be applied directly to either filling/orientation discrimination tasks (Experiment 1) or the large/small arrow tasks (Experiment 2).

However, previous studies suggest that numeral stimuli might contain spatial information, the so-called spatial-numerical association of response codes (Dehaene et al., 1993; Fischer, 2003). Participants associated digits smaller than five with left and numbers greater than five with right even though they were asked to apply the odd-even categorization rule in which magnitude was irrelevant (Dehaene et al., 1993). Hence it is possible that while the odd-even rule should have narrowed participants’ focus of attention toward the parity feature, the magnitude features may induce participants to also allocate attention to and perceive interference from the spatial feature: the direction of the arrows. This may have neutralized the shielding effect of the categorization rule. To avoid the spatial-numerical associations of response codes that may interfere with the results, future studies should replicate the results of Experiment 2 with different categorization rules.

## Conclusion

Consistent with the previous study (Li et al., 2019a), we found that bivalent features produced response-congruency effects even when participants do not know the bivalent features and related task rules yet. This suggests that bivalent feature-response associations may be formed passively. Moreover, we found that bivalent features that can easily be detected by the participants (e.g., two arrows) produced proactive interference and caused task-switching costs before we introduced bivalent features. Importantly, we also demonstrated the shielding function of the categorization rule and its potential limitation: Although categorization rules can shield certain interference away, strong interference from irrelevant features can break down the shielding function.

We would also argue that the limitation of the shielding function is a favorable characteristic of human cognitive control. Humans are alert to distractors because some distractors which might be irrelevant to the current task actually carry important information. Similar results have been observed and known as the cocktail party effect (Wood and Cowan, 1995): Listeners can easily attend to a single voice even when many people are talking simultaneously. Moreover, an auditory stimulus that is from an unattended speaker but highly pertinent to the listener, such as his/her name, can immediately capture the listener’s focus of attention.

## Data Availability

All datasets generated for this study are included in the manuscript and/or the supplementary files.

## Ethics Statement

Two experiments were carried out in accordance with the recommendations of Fudan University Department of Psychology Ethics Committee. All participants gave their written consents to take part into the experiments.

## Author Contributions

All authors listed have made a substantial, direct and intellectual contribution to the work, and approved it for publication.

## Conflict of Interest Statement

The authors declare that the research was conducted in the absence of any commercial or financial relationships that could be construed as a potential conflict of interest.

## Appendix

**Table A1:** Experiment 1.

Task/ Trial /Stage	Stage1		Stage2		Stage3	
	RT ms (*SD*)	ER% (*SD*)	RT ms (*SD*)	ER% (*SD*)	RT ms (*SE*)	ER% (*SD*)
*Association*						
Repeat con	689 (115)	2.34 (3.88)	655 (114)	1.65 (2.97)	978 (189)	1.89 (2.15)
Repeat inc	706 (123)	2.31 (3.41)	678 (126)	2.92 (3.78)	1014 (186)	6.64 (5.33)
Switch con	691 (111)	2.43 (3.22)	652 (111)	2.39 (2.79)	1149 (239)	4.04 (4.56)
Switch inc	717 (129)	3.11 (2.35)	687 (140)	4.00 (3.48)	1189 (242)	8.30 (5.14)
Repeat	697 (116)	2.35 (3.28)	666 (119)	2.32 (2.86)	995 (183)	4.33 (2.99)
Switch	704 (119)	2.79 (2.43)	669 (124)	3.12 (2.69)	1168 (238)	6.21 (4.13)
Congruent	690 (112)	2.44 (3.32)	653 (110)	2.02 (2.64)	1064 (201)	3.13 (3.23)
Incongruent	711 (125)	2.86 (2.20)	682 (132)	3.48 (3.08)	1104 (203)	7.52 (4.39)
Total	693 (114)	2.65 (2.63)	667 (121)	2.75 (2.59)	1083 (201)	5.32 (3.23)
*Numeral*						
Repeat con	612 (95)	3.62 (2.85)	607 (95)	3.32 (2.51)	872 (155)	5.18 (8.77)
Repeat inc	608 (83)	3.28 (2.92)	611 (99)	4.57 (3.24)	900 (152)	11.2 (10.62)
Switch con	606 (88)	3.81 (3.68)	619 (104)	3.93 (3.42)	1037 (216)	8.07 (8.14)
Switch inc	614 (93)	4.33 (3.26)	615 (100)	4.37 (3.38)	1088 (216)	16.51 (13.37)
Repeat	610 (88)	3.44 (2.26)	609 (97)	3.96 (2.20)	885 (149)	8.35 (9.10)
Switch	610 (90)	4.06 (3.09)	617 (101)	4.16 (3.00)	1059 (213)	12.11 (9.61)
Congruent	609 (90)	3.65 (2.76)	613 (99)	3.61 (2.06)	956 (183)	6.74 (8.80)
Incongruent	611 (88)	3.84 (2.37)	613 (99)	4.56 (2.69)	995 (178)	13.93 (11.25)
Total	603 (88)	3.73 (2.29)	613 (99)	4.08 (2.15)	975 (178)	10.34 (8.97)

*Mean (SD) of RT and ER for Each Trial Condition and Stage. Con = Congruent; Inc = Incongruent; Association = Association group; and Numeral = Numeral-categorization group.*

**Table A2:** Experiment 2.

Task/ Trial /Stage	Stage1		Stage2		Stage3	
	RT ms (*SD*)	ER% (*SD*)	RT ms (*SD*)	ER% (*SD*)	RT ms (*SE*)	ER% (*SD*)
*Association*						
Repeat con	728 (108)	1.88 (1.69)	679 (120)	3.01 (2.50)	724 (119)	0.46 (0.93)
Repeat inc	784 (124)	4.42 (2.90)	710 (121)	5.61 (3.68)	904 (188)	3.50 (3.11)
Switch con	748 (134)	2.23 (1.59)	697 (121)	2.69 (2.29)	815 (153)	2.00 (2.29)
Switch inc	812 (140)	6.39 (4.37)	742 (134)	4.85 (3.52)	991 (169)	8.38 (6.25)
Repeat	756 (113)	3.18 (1.86)	694 (118)	4.24 (2.58)	814 (144)	2.08 (1.72)
Switch	779 (131)	4.27 (2.52)	720 (126)	3.78 (2.45)	896 (148)	5.05 (3.50)
Congruent	739 (120)	2.10 (1.30)	689 (119)	2.85 (1.96)	771 (132)	1.26 (1.27)
Incongruent	800 (130)	5.47 (3.03)	728 (126)	5.08 (2.74)	946 (175)	6.10 (3.83)
Total	762 (117)	3.79 (1.90)	708 (121)	3.97 (2.08)	857 (143)	3.68 (2.13)
*Numeral*						
Repeat con	619 (94)	1.54 (1.59)	609 (86)	1.65 (1.87)	763 (170)	0.59 (0.96)
Repeat inc	651 (107)	3.88 (2.73)	636 (99)	5.00 (3.15)	969 (197)	5.59 (5.80)
Switch con	611 (109)	1.68 (2.04)	620 (102)	1.48 (1.63)	840 (214)	1.63 (1.44)
Switch inc	650 (98)	5.06 (3.90)	640 (98)	5.02 (3.56)	1061 (234)	7.95 (7.84)
Repeat	634 (98)	2.71 (1.67)	622 (89)	3.32 (2.13)	863 (171)	3.10 (3.15)
Switch	631 (101)	3.34 (2.14)	630 (97)	3.26 (1.87)	944 (209)	4.74 (3.95)
Congruent	614 (97)	1.62 (1.36)	615 (93)	1.56 (1.48)	803 (191)	1.14 (0.94)
Incongruent	651 (101)	4.42 (2.74)	638 (96)	4.99 (2.95)	1016 (212)	6.79 (6.14)
Total	626 (96)	3.02 (1.64)	627 (92)	3.28 (1.72)	906 (190)	3.97 (3.19)

*Mean (SD) of RT and ER for Each Trial Condition and Stage. Con = Congruent; Inc = Incongruent; Association = Association group; and Numeral = Numeral-categorization group.*

## References

[B1] Bogon J. Eisenbarth H. Landgraf S. Dreisbach G. (2017). Shielding voices: the modulation of binding processes between voice features and response features by task representations. *Q. J. Exp. Psychol.* 70 1856–1866. 10.1080/17470218.2016.1209686 27383254

[B2] Cooper P. S. Garrett P. M. Rennie J. L. Karayanidis F. (2015). Task uncertainty can account for mixing and switch costs in task-switching. *PLoS One* 10:e0131556. 10.1371/journal.pone.0131556 26107646 PMC4480360

[B3] Dehaene S. Bossini S. Giraux P. (1993). The mental representation of parity and number magnitude. *J. Exp. Psychol.* 122 371–396. 10.1037/0096-3445.122.3.371

[B4] Dreisbach G. (2012). Mechanisms of cognitive control. *Curr. Dir. Psychol. Sci.* 21 227–231.

[B5] Dreisbach G. Goschke T. Haider H. (2006). Implicit task sets in task switching? *J. Exp. Psychol. Learn. Mem. Cogn.* 32 1221–1233. 10.1037/0278-7393.32.6.1221 17087579

[B6] Dreisbach G. Goschke T. Haider H. (2007). The role of task rules and stimulus–response mappings in the task switching paradigm. *Psychol. Res.* 71 383–392. 10.1007/s00426-005-0041-3 16397812

[B7] Dreisbach G. Haider H. (2008). That’s what task sets are for: shielding against irrelevant information. *Psychol. Res.* 72 355–361. 10.1007/s00426-007-0131-5 18057961

[B8] Dreisbach G. Haider H. (2009). How task representations guide attention: further evidence for the shielding function of task sets. *J. Exp. Psychol. Learn. Mem. Cogn.* 35 477–486. 10.1037/a0014647 19271860

[B9] Dreisbach G. Wenke D. (2011). The shielding function of task sets and its relaxation during task switching. *J. Exp. Psychol. Learn. Mem. Cogn.* 35 477–486. 10.1037/a0024077 21707220

[B10] Fan J. McCandliss B. Sommer T. Raz A. Posner M. (2002). Testing the efficiency and independence of attentional networks. *J. Cogn. Neurosci.* 14 340–347. 10.1162/089892902317361886 11970796

[B11] Fischer M. (2003). Spatial representations in number processing: evidence from a pointing task. *Vis. Cogn.* 10 493–508. 10.1080/13506280244000186 27797538

[B12] Forrest C. L. Monsell S. McLaren I. (2014). Is performance in task-cuing experiments mediated by task set selection or associative compound retrieval? *J. Exp. Psychol. Learn. Mem. Cogn*. 40 1002–1024. 10.1037/a0035981 24564543

[B13] Friedman N. P. Miyake A. (2004). The relations among inhibition and interference control functions: a latent-variable analysis. *J. Exp. Psychol.* 133 101–135. 10.1037/0096-3445.133.1.101 14979754

[B14] Holm S. (1979). A simple sequentially rejective multiple test procedure. *Scan. J. Stat.* 6 65–70.

[B15] Hommel B. (1998). Event files: evidence for automatic integration of stimulus-response episodes. *Vis. Cogn.* 5 183–216. 10.1080/713756773

[B16] Hommel B. (2004). Event files: feature binding in and across perception and action. *Trends Cogn. Sci.* 8 494–500. 10.1016/j.tics.2004.08.007 15491903

[B17] Hommel B. (2005). Feature integration across perception and action: event files affect response choice. *Psychol. Res.* 71 42–63. 10.1007/s00426-005-0035-1 16341545

[B18] Kiesel A. Steinhauser M. Wendt M. Falkenstein M. Jost K. Philipp A. M. (2010). Control and interference in task switching: a review. *Psychol. Bull.* 136 849–874. 10.1037/a0019842 20804238

[B19] Li B. Li X. Liu X. Lages M. Stoet G. (2019a). Target-response associations can eliminate task-switching costs but not response congruency effects. *Front. Psychol.* 10:40. 10.3389/fpsyg.2019.00040 30804824 PMC6378947

[B20] Li X. Li B. Liu X. Lages M. Stoet G. (2019b). Task-switching costs disappear if non-Chinese participants respond to Chinese characters. *Exp. Psychol. Learn. Mem. Cogn.* 10.1037/xlm0000692 [Epub ahead of print]. 30730179

[B21] Mackie M. A. Van Dam N. T. Fan J. (2013). Cognitive control and attentional functions. *Brain Cogn.* 82 301–312. 10.1016/j.bandc.2013.05.004 23792472 PMC3722267

[B22] MacLeod C. (1991). Half a century of research on the stroop effect: an integrative review. *Psychol. Bull.* 109 163–203. 10.1037//0033-2909.109.2.163 2034749

[B23] Meier C. Lea S. E. McLaren I. P. (2016). Task-switching in pigeons: associative learning or executive control? *J. Exp. Psychol. Anim. Learn. Cogn.* 42 163–176. 10.1037/xan0000100 27054382

[B24] Meiran N. (2014). “The task-cueing paradigm: a user’s guide,” in *Task Switching and Cognitive Control* eds Grange J. Houghton G. (New York, NY: Oxford University Press) 45–73.

[B25] Meiran N. Kessler Y. (2008). The task rule congruency effect in task switching reflects activated long-term memory. *J. Exp Psychol. Hum. Percept. Perform.* 34 137–157. 10.1037/0096-1523.34.1.137 18248145

[B26] Pettigrew C. Martin R. C. (2014). Cognitive declines in healthy aging: evidence from multiple aspects of interference resolution. *Psychol. Aging* 29 187–204. 10.1037/a0036085 24955989

[B27] R Core Team (2017). *R: A Language and Environment for Statistical Computing.* Vienna, Austria: R Foundation for Statistical Computing. Available at: https://www.R-project.org/ 10.1037/a0036085 (accessed December 20, 2018).

[B28] Reisenauer R. Dreisbach G. (2013). The impact of task rules on distracter processing: automatic categorization of irrelevant stimuli. *Psychol. Res.* 77 128–138. 10.1007/s00426-012-0413-4 22252305

[B29] Reisenauer R. Dreisbach G. (2014). The shielding function of task rules in the context of task switching. *Q. J. Exp. Psychol. Hum. Exp. Psychol.* 67 358–376. 10.1080/17470218.2013.808678 23805948

[B30] Schneider D. Logan G. (2009). Selecting a response in task switching: testing a model of compound cue retrieval. *J. Exp. Psychol. Learn., Mem. Cogn.* 35 122–136. 10.1037/a0013744 19210085 PMC2667949

[B31] Schneider D. W. Logan G. D. (2014). Modelling response selection in task switching: testing the contingent encoding assumption. *Q. J. Exp. Psychol.* 67 1074–1095. 10.1080/17470218.2013.843009 24138405 PMC4315513

[B32] Schneider D. W. (2015). Isolating a mediated route for response congruency effects in task switching. *J. Exp. Psychol. Learn. Mem. Cogn.* 41 235–245. 10.1037/xlm0000049 25068859

[B33] Schneider D. W. (2018). Categorization difficulty modulates the mediated route for response selection in task switching. *Psychon. Bull. Rev.* 25 1958–1967. 10.3758/s13423-017-1416-3 29274057

[B34] Stoet G. (2010). PsyToolkit: a software package for programming psychological experiments using Linux. *Behav. Res. Methods* 42 1096–1104. 10.3758/BRM.42.4.1096 21139177

[B35] Stoet G. (2017). PsyToolkit. *Teach. Psychol.* 44 24–31. 10.3758/BRM.42.4.1096 21139177

[B36] Sudevan P. Taylor D. (1987). The cueing and priming of cognitive operations. *J. Exp. Psychol. Hum. Percept. Perform.* 13 89–103. 2951490 10.1037//0096-1523.13.1.89

[B37] Vandierendonck A. Liefooghe B. Verbruggen F. (2010). Task switching: interplay of reconfiguration and interference control. *Psychol. Bull.* 136 601–626. 10.1037/a0019791 20565170

[B38] Weinbach N. Henik A. (2014). Altering enhances attentional bias for salient stimuli: evidence from a global/local processing task. *Cognition* 133 414–419. 10.1016/j.cognition.2014.07.005 25128799

[B39] Wendt M. Kiesel A. (2008). The impact of stimulus-specific practice and task instructions on response congruency effects between tasks. *Psychol. Res.* 72 425–432. 10.1007/s00426-007-0117-3 17546462

[B40] Wood N. L. Cowan N. (1995). The cocktail party phenomenon revisited: attention and memory in the classic selective listening procedure of Cherry (1953). *J. Exp. Psychol. Gen.* 124 243–262. 10.1037/0096-3445.124.3.243 7673862

[B41] Woodward T. Meier B. Tipper C. Graf P. (2003). Bivalency is costly: bivalent stimuli elicit cautious responding. *Exp. Psychol.* 50 233–238. 10.1026//1618-3169.50.4.233 14587170

